# 598. Hot Spots: Zonal Cutaneous Functional Unit Analysis of High-Risk Regions in Pediatric Shoulder/Hip Scald Burns

**DOI:** 10.1093/jbcr/irag033.402

**Published:** 2026-04-06

**Authors:** Christian Hudson-Bradford, Alexander J Kallabat, Candace Marquette, Elika Ridelman, Beth A Angst, Sarah Hackert, Justin D Klein, Christina M Shanti

**Affiliations:** Children's Hospital - Michigan, Detroit, MI; Wayne State University School of Medicine, Bloomfield Hills, MI; Wayne State University School of Medicine, Detroit, MI; Wayne State University School of Medicine, Children's Hospital of Michigan Burn Center, Detroit, MI; Children's Hospital - Michigan, Sterling Heights, MI; Children's Hospital - Michigan, Sterling Heights, MI; Wayne State University School of Medicine, Children's Hospital of Michigan Burn Center, Detroit, MI; Wayne State University School of Medicine, Children's Hospital of Michigan Burn Center, Detroit, MI

## Abstract

**Introduction:**

Pediatric scald burns present significant healing challenges. This study utilizes a novel zonal classification of cutaneous functional units (CFUs) of the shoulder and hip to identify anatomical risk factors and patient characteristics associated with poor outcomes, specifically delayed healing (>14 days) and the need for scar management (SM).

**Methods:**

A retrospective review was performed on a cohort of 967 pediatric scald burn patients (0-18 years) admitted to a regional burn center from 2015–2024. An expert panel mapped burn photographs to a six-zone anatomical classification of the shoulders and hips using standardized diagrams. Chi-square tests, ANOVA, and t-tests were used to analyze links between patient demographics, burn characteristics, specific zones, and outcomes. Outcomes were defined as time to complete healing (TTH, in days) and the need for SM, defined as treatment requiring at least silicone tape, compression garments, steroid injections, or laser therapy.

**Results:**

Among 736 patients with complete healing data, delayed healing strongly predicted need for SM (41.6% of TTH >28 days required SM vs 2.9% of ≤14 days, *p*<.001). Factors predicting prolonged TTH included hot oil/grease etiology (*p*=.018), need for an index graft (*p*<.001), and ICU admission (*p*<.001). Bilateral shoulder (*p*=.027) and hip (*p*=.005) involvement were also significantly associated with prolonged healing. Zone-level analysis revealed distinct anatomic risk patterns: posterior-inferior hip zone 6 healed slowest (median 31 days) with the highest SM rates (32%, *p*<.05 vs multiple other hip zones). Hip zone 5 (median 21.5 days, 17% SM) and hip zone 3 (median 22 days, 17.4% SM) also demonstrated delayed healing compared to anterior zones. Shoulder zone 1 (anterior) consistently healed faster (median 15 days) than zones 2–5 (*p*≤.05), while zones 3 and 5 carried the greatest scar burden (>18% SM).

The need for SM was also significantly higher in patients with an index graft (83.3% vs 11.3%, *p*<.001), post-burn complications (64.0% vs 11.3%, *p*<.001), and burn-related readmissions (61.3% vs 10.8%, *p*<.001). Surprisingly, Fitzpatrick score did not show any association with SM need.

**Conclusions:**

This zonal CFU analysis identifies posterior-inferior hip zone 6 as the most vulnerable region, with healing delays exceeding 30 days and severe scarring in up to one-third of patients. Additional high-risk zones include hip zones 3 and 5 and shoulder zones 3 and 5, which consistently demonstrated worse outcomes. Conversely, shoulder zone 1 healed significantly faster with no added scar risk.

**Applicability of Research to Practice:**

These findings suggest that clinicians should prioritize early use of silicone taping, compression garments, laser treatments, and other scar-prevention strategies for posterior hip and shoulder burns compared to the anterior regions. Zonal mapping thus provides anatomic precision to guide risk stratification and proactive intervention in pediatric scald burns.

**Funding for the study:**

Foundation Funding.

